# *Erwinia amylovora* bacteriophages as biological agents for targeted and sustainable fire blight management

**DOI:** 10.3389/fpls.2026.1890132

**Published:** 2026-08-18

**Authors:** Anđelka Prokić, Milan Ivanović, Nemanja Kuzmanović, Nevena Zlatković, Jelena Adamović, Nebojša Nedić, Aleksa Obradović, Katarina Gašić

**Affiliations:** 1 Department of Phytopathology, Institute of Phytomedicine, University of Belgrade – Faculty of Agriculture, Belgrade, Serbia; 2 Institute for Plant Protection in Horticulture and Urban Green, Julius Kühn Institute (JKI) - Federal Research Centre for Cultivated Plants, Braunschweig, Germany; 3 Department of Plant Diseases, Institute for Plant Protection and Environment, Belgrade, Serbia

**Keywords:** apple, bacteriophages, biocontrol, *Erwinia amylovora*, fire blight, pear, phage therapy

## Abstract

Fire blight, caused by the Gram-negative bacterium *Erwinia amylovora*, is one of the most destructive diseases of pome-fruit crops worldwide. Despite decades of research, its management remains challenging because efficient and sustainable control options are limited, and many commercially important apple and pear cultivars remain highly susceptible. Conventional treatments for bacterial disease suppression are often hindered by inconsistent efficacy, phytotoxic effect, environmental concerns and regulatory restrictions, necessitating new sustainable alternative options. Virulent bacteriophages have emerged as promising biocontrol agents for controlling *E. amylovora* due to their unique biological properties, including a distinct mode of action, capacity to replicate within bacterial cells and high host specificity. These features enable effective suppression of pathogen populations, while potentially minimizing impact on non-target microbiota, depending on their host range and ecological interactions. However, further research is needed to better understand phage-host interactions, safety issues, and the environmental factors that influence phage efficacy in field application. Furthermore, combining phages with other compatible management approaches may improve disease control and help mitigate the emergence of resistant pathogen populations. This narrative review summarizes the key characteristics of *E. amylovora* phages, and recent advances in phage-based fire blight control, highlighting their potential role in integrated and sustainable disease management strategies.

## 1 Introduction

### 1.1 *Erwinia amylovora* distribution, epidemiology and impact

*Erwinia amylovora* is a widely distributed, Gram-negative, facultatively anaerobic, plant-pathogenic bacterium belonging to the family *Erwiniaceae* within the order *Enterobacterales* (Burrill, 1882; Winslow et al., 1920; van der Zwet, 2006; Adeolu et al., 2016). It is the causal agent of fire blight (FB), one of the most destructive bacterial diseases, affecting more than 200 plant species from the family *Rosaceae*, including pome-fruit crops, wild trees and ornamentals (Vanneste, 2000). The most economically important hosts are apple (*Malus domestica*), pear (*Pyrus communis*) and quince (*Cydonia oblonga*) (Momol and Aldwinckle, 2000; van der Zwet, 2012). Despite the absence of formally recognized biovars or pathovars, *E. amylovora* populations can be divided into two genetically distinct groups, with respect to their preferred hosts (Zeng et al., 2018; Parcey et al., 2020; Mendes et al., 2024).

Native to North America, where it was first discovered at the end of the 18th century by Thomas J. Burrill, FB was introduced into Europe in the 1950s. Since then, the disease has spread to over 60 countries worldwide, threatening global pome-fruit production (European and Mediterranean Plant Protection Organization, 2025; Park et al., 2018). In recent years, FB has also been reported in parts of Central and East Asia, including Kyrgyzstan, Kazakhstan, South Korea and China (Park et al., 2016; Sun et al., 2023). Its spread into previously unaffected areas are promoted by intensive crop production, international trade of planting material, the use of susceptible cultivars and rootstocks, climate change and limited availability of consistently effective disease-management options (Gayder et al., 2023). In the European Union (EU), *E. amylovo*ra is regulated under Commission Implementing Regulation 2019/2072 (European Commission, Directorate-General for Health and Food Safety, SANTE, 2019). Outside designated protected zones it is listed as a regulated non-quarantine pest (RNQP) for specified host plants and plant commodities.

FB can cause severe regional economic losses in pome-fruit production. The extent of damage depends on cultivar susceptibility, climate conditions, inoculum pressure, orchard age and disease management practices. In both the United States (US) and Europe, severe outbreaks have resulted in costly tree removal, intensified disease management and, in some cases, replanting; emphasizing the need for sustainable and cost-effective control options (Norelli et al., 2003; Zhao et al., 2019; Gayder et al., 2023).

The disease cycle begins with an epiphytic phase, during which *E. amylovora* colonizes host plant surfaces and proliferates on floral stigmas, where cells can form a biofilm matrix that protects them from environmental stress-factors. Resident bacteria subsequently enter plant tissues through natural openings, mainly flower nectarthodes, or through wounds (Thompson, 2000). Primary infections commonly occur during bloom and may continue around petal fall, when environmental conditions favor bacterial multiplication and flower infection. Under optimal conditions, *E. amylovora* spreads systemically through the host plant, moving though intracellular spaces in parenchyma tissues, reaching the xylem vessels at latter stages. It can infect all parts of the tree, including flowers, leaves, shoots, fruits, branches, trunk tissues and rootstock (Geider, 2000; Vanneste and Eden-Green, 2000; Koczan et al., 2011; Castiblanco and Sundin, 2016).

As a result of bacterial activity, infected tissues become desiccated and wilted, accompanied by blackening and scorched appearance of affected foliage, which may progress to partial or complete blighting and necrosis of the canopy. Typical disease symptom, arising from bacterial colonization of shoots, xylem, and parenchyma tissues, is the formation of a “shepherd’s crook” at the shoot tips. Canker wounds that develop on branches, twigs and trunk can produce amber-colored bacterial ooze, which contains high populations of bacterial cells embedded in a polysaccharide-rich matrix. This exudate is an important source of inoculum and facilitates pathogen dissemination by insects and wind-driven rain (Vanneste and Eden-Green, 2000). Under favorable climatic conditions, FB outbreaks can result in the loss of individual trees or even entire orchards (van der Zwet and Keil, 1979; Bonn and van der Zwet, 2000).

*E. amylovora* employs multiple virulence factors that promote host colonization, immune suppression, and disease development, including extracellular polymers (exopolysaccharides, EPSs), the type III secretion system (T3SS), motility, biofilm formation and quorum sensing (Koczan et al., 2011; Piqué et al., 2015). The production of copious amounts of two principal EPS, amylovoran and levan, is essential for pathogenicity and virulence, contributing to host-cell interactions and plant colonization, biofilm formation within xylem vessels, antimicrobial and plant defense resistance and symptom development. *E. amylovora* also uses flagellar motility to move from stigma tips to the hypanthia and through nectar. Flagella have been suggested to play an important role in virulence (Schachterle et al., 2022). In addition, small ribonucleic acid molecules (sRNAs) modulate plant–pathogen interactions in *E. amylovora* by regulating EPS production, surface attachment, swimming motility and virulence gene expression during infection (Zeng et al., 2013; Zeng and Sundin, 2014).

### 1.2 Disease management strategies

Despite a long history of research, FB remains a major concern in pome-fruit production due to lack of efficient and sustainable control strategies and susceptibility of commercially desirable apple and pear cultivars. In most regions, FB management relies on an integrated approach that combines preventive phytosanitary measures, chemical and biological control agents, disease forecasting systems and cultural practices (Norelli et al., 2003; Johnson et al., 2016; van der Zwet and Beer 1999; Zhao et al., 2019; Wallis and Cox, 2020; Zeng et al., 2023).

In the US, the most effective FB control was achieved through prophylactic application of antibiotic-based bactericides, including streptomycin, oxytetracycline and kasugamycin, during bloom period, when primary infection occurs (Jones and Schnabel, 2000; Mc Ghee and Sundin 2011; Mc Manus, 2014; McManus and Stockwell, 2001; Psallidas and Tsiantos, 2000; Svircev et al., 2018). By contrast, chemical treatments for controlling shoot and rootstock blight during summer are generally less effective, largely because infections are already established within plant tissues.

However, extensive use of streptomycin has led to selection of streptomycin-resistant *E. amylovora* strains in several US countries, resulting in reduced disease control efficacy (Loper et al., 1991; McManus and Jones, 1994; McManus et al., 2002; Russo et al., 2008; Stockwell and Duffy, 2012; Tancos et al., 2016; Slack et al., 2021). Moreover, transfer of this resistance is readily achieved through acquisition of mobile genetic elements such as plasmids, further complicating long-term disease management (Sundin et al., 2023).

In Europe, regulatory restrictions on antibiotic use in plant protection have led to predominant use of copper-based bactericides, which can effectively reduce primary inoculum (Duffy et al., 2005; Sundin and Wang, 2018). However, due to the phytotoxic effects, copper application is largely limited to winter and pre-flowering periods, leaving growers with few effective control options during the most susceptible stage of infection. Taken together, the limited efficacy of conventional disease management, the emergence and spread of copper- and antibiotic-tolerant strains, disruption of orchard ecosystems, and increasing consumer demand for pesticide-free products, have driven the search for more sustainable alternatives. Biological control agents (BCAs) represent a promising and environmentally friendly strategy for managing infections caused by *E. amylovora*, while also addressing concerns associated with the excessive antibiotic and copper use in agriculture (Johnson and Stockwell, 2000; Nagy et al., 2012; Gayder et al., 2023).

Bacteriophage (phage) therapy, which involves the application of viruses that specifically infect and lyse bacterial cells, represents a highly specific biological approach for the control of bacterial plant disease, including FB (Balogh et al., 2010; Jones et al., 2012; Schnabel et al., 1998). This narrative review synthesizes published studies on the isolation, biological and genomic characterization, formulation and biocontrol efficacy of *E. amylovora*-targeting phages, with emphasis on their potential use in integrated FB management.

Literature was identified through searches of Web of Science, Scopus, PubMed, Google Scholar, ICTV resources, EPPO and EU regulatory sources and publisher databases using combinations of the terms *Erwinia amylovora*, fire blight, bacteriophage, phage therapy, biocontrol, phage cocktail, formulation, resistance, host range, *Caudoviricetes* and phage biopesticide. Priority was given to peer-reviewed publications on *E. amylovora* phages, phage-based biocontrol, greenhouse or field efficacy, formulation and relevant taxonomy updates. Because the aim of this review was narrative synthesis rather than exhaustive evidence grading, no PRISMA-style screening or formal risk-of-bias assessment was performed.

## 2 Biological and ecological characteristics of *E. amylovora* bacteriophages

Bacteriophages (phages) are viruses that can specifically infect and destroy bacterial cells. They were discovered more than a century ago independently by Twort (1915) and d’Herelle (1917). As some of the most abundant and diverse biological entities on Earth, they play fundamental roles in ecosystems by shaping abundance, composition, dynamics and diversity of bacterial communities (Clokie et al., 2011). Although many phages exhibit a relatively narrow host range, host specificity must be determined empirically for each candidate phage, because it depends on the interaction between phage adsorption structures and bacterial surface receptors (Hyman and Abedon, 2010). This high specificity for certain bacteria, together with their capacity to replicate in target bacterial cells, makes them promising alternatives or complements to conventional pesticides used in agriculture and food safety (Balogh et al., 2010; Buttimer et al., 2017).

The first *E. amylovora*-specific phages were isolated in the 1950s, and their FB control potential was first studied in 1970s (Erskine, 1973; Ritchie and Klos, 1977; Ritchie and Klos, 1979). Since then, diverse *E. amylovora*-infecting phage strains with different infection strategies, physiological and genomic features have been isolated and characterized (Born et al., 2011; Boule et al., 2011; Müller et al., 2011; Nagy et al., 2015; Meczker et al., 2014; Yagubi et al., 2014; Esplin et al., 2017; Akremi et al., 2020; Park et al., 2022; Sabri et al., 2022; Hassan et al., 2023; Zlatohurska et al., 2023; Kim et al., 2024; Xi et al., 2024; Lomadze et al., 2025; Bouazizi et al., 2025). Multiple studies have demonstrated their potential as effective BCAs, emphasizing their specificity, ability to reduce disease severity in planta, and improved efficacy when applied combined as phage cocktails (Lehman, 2007; Lehman et al., 2009; Balogh et al., 2010; Nagy et al., 2012; Gašić et al., 2014, 2016; Yagubi et al., 2014; Buttimer et al., 2018; Gayder et al., 2023; Biosca et al., 2024; Ke et al., 2024; Kim et al., 2024) (**Table 1**).

**Table 1:** Overview of studied *E. amylovora* phages from 2003-2025.

Phage (s) designation(s)	Geographical origin	Historical morphotype	Current ICTV taxonomy	Isolation source	Genome size (kb)	Type of study	Reference
PEa10 (16 isolates), PEa51 (6 isolates)	Canada	PEa10: Myovirus-like and(Podovirus-like), PEa51: Podovirus-like	class Caudoviricetes	trees and soil	/	Isolation, diversity	Gill et al. (2003)
Ea2345-6, Ea2345-19, Ea1337-26	Canada	Ea2345-6, Ea2345-19: Myovirus-like, Ea1337-26: Podovirus-like	class Caudoviricetes	soil	74, 75, 36	isolation, characterization; in planta biocontrol efficacy	Boulé et al. (2011)
L1, M7, and Y2 vB_EamP-S2, S6, vB_EamM-Bue1	Switzerland	L1, S6: Podovirus-like, M7, Y2: Myovirus-like	class Caudoviricetes; L1: family Autographiviridae, genus Pectivirus, S6: family Schitoviridae, genus Enquatrovirus,: M7: family Ackermannviridae, genus Kuttervirus,Y2: family Chaseviridae, genus Y2 virus,	Pome-fruit orchards	39.3 (L1), 74. 7 (S6), 84.7 (M7), and 56.6 (Y2),	isolation, genome sequencing, phage cocktail efficacy *in vitro*	Born et al. (2011); Knecht et al. (2018)
Ea1h, Ea100, Ea104	North America	Ea1h, Ea100: Podovirus-like, Ea104: Myovirus-like	class Caudoviricetes, Ea1h, Ea100: family Autosignataviridae, subfamily Molineuxvirinae, genus Eracentumvirus and Ea104: family Andersonviridae, subfamily Ounavirinae, genus Kolesnikviruss	apple orchards	45. 5;45. 5; 84. 5	genomic information	Müller et al. (2011)
ΦEa1, ΦEa2, ΦEa3, ΦEa4, ΦEa5, Ea6, ΦEa7	Serbia	Myovirus-like and Siphovirus-like	class Caudoviricetes	rhizosphere, irrigation water pear and apple leaves	/	characterization, biocontrol in detached blossom assay	Gasić et al. (2014)
PhiEaH1	Hungary	Siphovirus-like	class Caudoviricetes, family Demerecviridae, genus EaH1virus	soil	218. 3	genomic information	Meczker et al. (2014)
Ea35-70	Canada	Myovirus-like	class Caudoviricetes	soil	271. 1	genome sequence	Yagubi et al. (2014)
phiEa2809	Belarus	Myovirus-like	class Caudoviricetes, family Ackermannviridae, genus Nezavisimistyvirus (species: Nezavisimistyvirus Ea2809)	symptomless apple leaf	162. 1	characterization	Lagonenko et al. (2015)
H5K	Hungary	Myovirus-like	class Caudoviricetes	quince shoots and flowers	N/A	in planta translocation	Nagy et al. (2015)
phiEaP-8	South Korea	Podovirus-like	class Caudoviricetes, family Schitoviridae, subfamily Erskinevirinae, genus Yonginvirus	soil	75. 9	characterization	Park et al. (2018)
pEa_SNUABM_12, pEa_SNUABM_47, and pEa_SNUABM_50	South Korea	Myovirus-like	class Caudoviricetes,genus Eneladusvirus	water and soil	360	isolation, characterization, genomic analysis	Kim et al. (2020)
PEar1, PEar2, PEar4 and PEar6	Tunisia	Filamentous	class Faserviricetes, order, Tubulavirales, family, Inoviridae, genus Lineavirus	soil and pear tissue	6. 6- 6. 8	characterization	Akremi et al. (2020)
ΦFifi: 011, 044, 051, 067, 106, 287, 318, 450, 451	South Korea	Podovirus-like	class Caudoviricetes, family Chaseviridae, genus Fifivirus	soil and water samples	53.4-84.5	characterization and host range	Park et al. (2022); Choe et al. (2023)
EP-IT22	Italy	Myovirus-like	class Caudoviricetes, family Straboviridae, genus Cronusvirus	wastewater	174. 3	characterization, biocontrol in planta	Sabri et al. (2022)
EAP1, EAP2, EAP3	Egypt	EAP1, EAP2: Siphovirus-like, EAP3:Myovirus-like	class Caudoviricetes	pear trunk and soil	N/A	characterization	Hassan et al. (2023)
Key	Ukraine	Siphovirus-like	class Caudoviricetes, family Demerecviridae, genus Keyvirus	quince	115.6	genome characterization	Zlatohurska et al. (2023)
ΦFifi 044, ΦFifi 318	South Korea	Myovirus-like		water (pear), water (apple)	N/A	phage cocktail efficacy in planta	Kim et al. (2024)
RH-42-1	China	Tectivirus	class Tectiliviricetes family Tectiviridae, genus Alphatectivirus	soil	14. 9	characterization	Xi et al. (2024)
Hena1, Hena 2, Roscha1, Pixel, Dichka, VyarbaS, Vyarba L, Fleur, Micant, Lohitsa1, Loshitsa 2, Stepyanka	Belarus	Hena1, Hena2, Roscha1, Pixel, Dichka, VyarbaS: Myovirus-like, VyarbaL, Loshitsa1, Loshitsa2, Micant, Fleur, Stepyanka: Podovirus-like	class Caudoviricetes; families Chimalliviridae (Hena1, Hena 2), Autographiviridae (Stepyanka)	soil	39. 3-148.8	characterization	Besarab et al. (2020; 2021; 2023)
Eaφ1-Eaφ28	Spain	Myovirus-like	class Caudoviricetes, family Andersonviridae, genus Kolesnikvirus	pear tree and soil	~84. 7	characterization, phage cocktail efficacy in planta	Biosca et al. (2024)
Omen	Portugal	Myovirus-like	class Caudoviricetes, family Andersonviridae, subfamily Ounavirinae, genus Kolesnikvirus	soil	85. 3	genome analysis	Abreu et al. (2024)
phiEaSP1	South Korea	Podovirus-like	class Caudoviricetes, family Chaseviridae, genus Loessnervirus	water	53. 9	characterization, phage cocktail efficacy in planta	Kim et al. (2025)
EAP2, EAP4	Turkey	Myovirus-like	class Caudoviricetes	soil	85. 7, 86. 2	genomic and biological characterization	Evran et al. (2025)
pHEa1, pHEa2, pHEa3, pHEa4	Tunisia	pHEa1, pHEa2: Myovirus-like, pHEa3, pHEa4: Podovirus-like	class Caudoviricetes	pear leaves, soil	N/A	characterization, phage cocktail efficacy in planta	Bouazizi et al. (2025)
EaPF7, EaPF9	Hungary	Myovirus-like	class Caudoviricetes, family Chaseviridae, genus Loessnervirus	soil	54. 1, 52. 9	characterization	Lomadze et al. (2025)

### 2.1 Habitat and isolation sources

Phage populations closely accompany the development of bacterial communities within ecosystem. Consequently, orchards infected by FB provide most suitable ecological niches for the isolation of *E. amylovora*-specific phages. However, they can also be recovered from a variety of other sources, including decaying organic matter, compost, and plant debris - niches where the bacterial host is likely to persist (Gill et al., 2003; Gayder et al., 2023). Such environments serve as natural reservoirs, supporting the coexistence of phages and their bacterial hosts. Environmental diversity increases the probability of recovering genetically and biologically diverse phages, while repeated sampling throughout the growing season may further enhance diversity by capturing temporal fluctuations in phage populations (Kaneko et al., 2026).

Most *E. amylovora* phages have been isolated from the rhizosphere of infected plants, where soil conditions provide protection against desiccation and ultraviolet (UV) radiation, both of which can adversely affect phage viability (Erskine, 1973; Williams et al., 1987; Schnabel and Jones, 2001; Balogh, 2002; Iriarte et al., 2007; 2012). Phages have also been detected in the phyllosphere, including infected blossoms, leaves and branches, where they may directly interact with bacterial populations. In addition, they have been recovered from irrigation water and vegetation surrounding infected orchards, while a smaller proportion has been isolated from infected aerial tissues of pear and apple trees (Gill et al., 2003; Gašić et al., 2014). To maximize the recovery of a more diverse phage population with a broader host range, multiple bacterial host strains from different sources should be used during isolation, rather than relying on single-host selection (Xi et al., 2024; Bouazizi et al., 2025). This approach is consistent with the findings of Gill et al. (2003) who increased the diversity of recovered phages by using six different bacterial hosts during the initial isolation and enrichment processes.

### 2.2 Taxonomy and morphology characteristics

Accurate and comprehensive characterization and classification of phages are essential for understanding their ecological roles and the development of effective phage-based control strategies (Ackermann and Eisenstark, 1974). Phage isolates can be characterized and differentiated into distinct subtypes using plaque morphology, host range, pulsed-field gel electrophoresis (PFGE) and PCR-based analysis (Schnabel and Jones, 2001; Gill et al., 2003; Born et al., 2011).

Originally, phage isolates were classified based primarily on virion morphology observed by transmission electron microscopy (TEM) in distinct morphotypes Ackerman and Eisenstark, 1974; **Table 2**. With the increasing availability of complete genome sequences, this approach has been complemented by more detailed and comprehensive genome-based characterization (Turner et al., 2021). In alignment with current ICTV (International Committee on Taxonomy of Viruses), phage taxonomy is based on polyphasic approach that integrates genome sequences, protein similarity and molecular phylogeny, while morphology-based groups have been abolished (Ackermann and Prangishvili, 2012; ICTV, 2025). Therefore, some historical isolates that have not been genomically characterized remain unclassified within the class *Caudoviricetes*. In addition, the descriptive terms myovirus-like, siphovirus-like and podovirus-like, remain useful for describing virion structure (Walker et al., 2022; Simmonds et al., 2023; Turner et al., 2023; 2025). In this review, these terms are used only as morphological descriptors, whereas formal ICTV taxonomic names are provided where available.

**Table 2:** Structural features of *E. amylovora* phages genome-delivery tail machine.

Feature	Myovirus-like	Podovirus-like	Siphovirus-like
Tail length	Long (100–140 nm)	Very short (10–20 nm)	Long, flexible (100–200 nm)
Tail type	Contractile	Non-contractile	Non-contractile
Genome-delivery	Mechanical force via sheath contraction	Pressure-driven via internal core proteins	Passive + enzymatic
Baseplate	Large, multi-arm (T4-like)	Compact (T7-like)	Simple
Injection tube	Rigid tail tube	Transient proteinaceous channel	Tail tube + minor proteins
Genome size	140–170 kb	~40 kb	~50–60 kb
Complexity	Highest	Moderate	Moderate

Morphotype names are descriptive and should not be interpreted as current formal ICTV family names.

**Figure 1:**
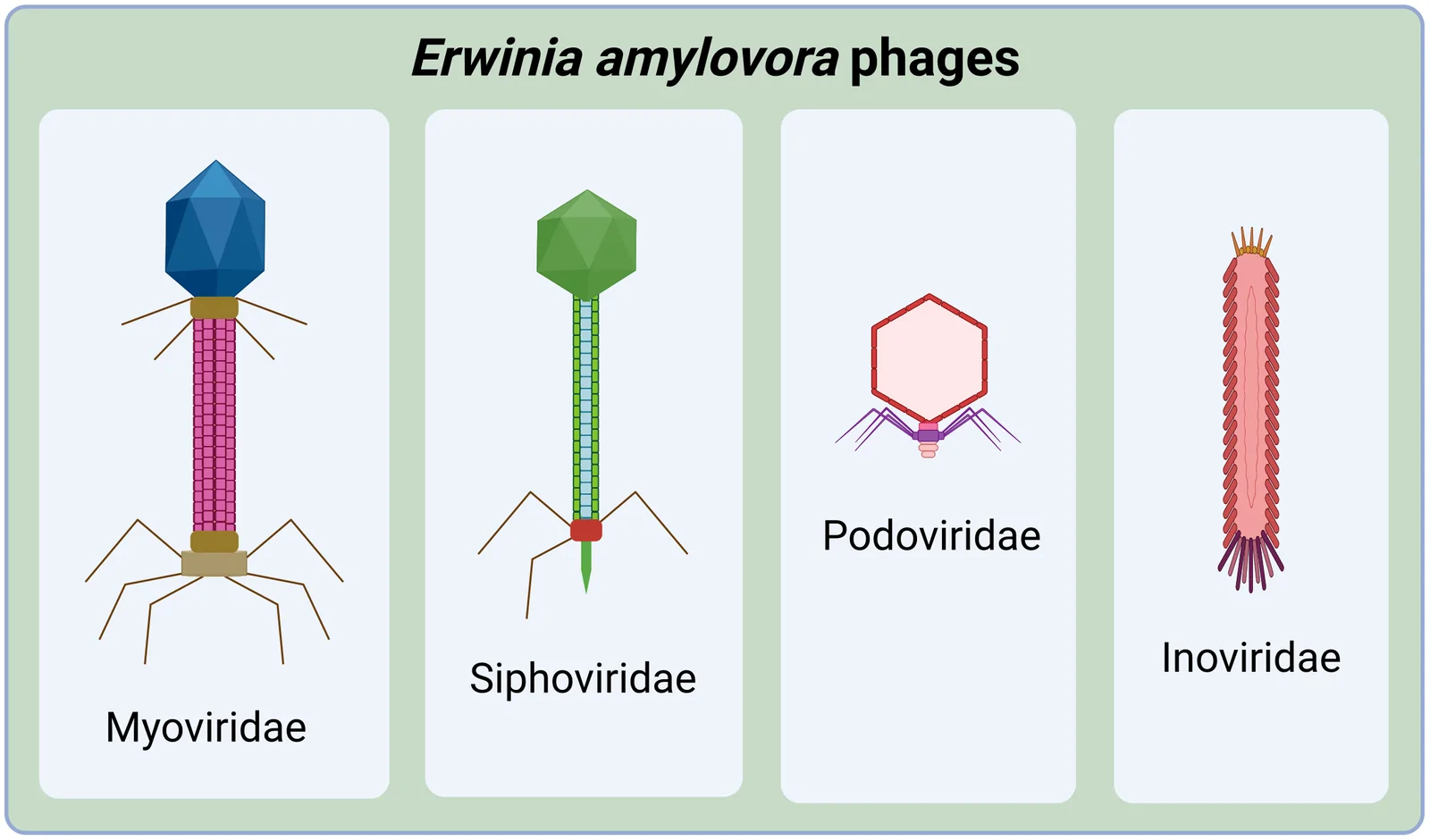
Illustrative scheme showing particle morphology of *E. amylovora* bacteriophages Myovirus-like, siphovirus-like and podovirus-like refer to virion morphotypes and are not used here as current formal ICTV family names. Created in BioRender.

Diverse *E. amylovora* phages have been reported to date, mostly with icosahedral head, tail and double-stranded DNA (dsDNA) structure (**Table 1**). These isolates were originally assigned within the order *Caudovirales* and paraphyletic families *Myoviridae*, *Podoviridae* and *Siphoviridae* (**Figure 1**). Phages with myovirus- and podovirus-like morphology, characterized by long, contractile and short, non-contractile tails, respectively, represent an important component of *E. amylovora* phage diversity. They have been frequently recovered in Canada (Gill et al., 2003; Boulé et al., 2011; Yagubi et al., 2014), Belarus (Lagonenko et al., 2015; Besarab et al., 2020; 2021; 2023), Switzerland (Born et al., 2011), US and Germany (Müller et al., 2011a, 2011b; Nagy et al., 2015), Serbia (Gašić et al., 2014; 2016), Hungary (Nagy et al., 2015; Lomadze et al., 2025), South Korea (Kim et al., 2020; 2024; 2025; Park et al., 2018; 2022; Choe et al., 2023), Italy (Sabri et al., 2022), Egypt (Hassan et al., 2023), Spain (Biosca et al., 2024), Portugal (Abreu et al., 2024). *E. amylovora* phages with siphovirus-like morphology, with long, flexible and non-contractile tails appear to be less frequently found (Meczker et al., 2014; Zlatohurska et al., 2023; Ke et al., 2024; Evran et al., 2025).

Recent genomic studies have revealed substantial taxonomic and biological diversity among *E. amylovora* phages, providing higher resolution in assessing gene content and biocontrol suitability (Müller et al., 2011; Dömötör et al., 2012; Lagonenko et al., 2015; Esplin et al., 2017; Knecht et al., 2018; Sharma et al., 2018; Thompson et al., 2019; Abreu et al., 2024). The first known *E. amylovora* phage with tadpole-shaped architecture (icosahedral capsid with internal lipid membrane and short tail-like non-contractile vertex) was isolated from FB–affected orchards in China (Xi et al., 2024). Genomic and phylogenetic analyses demonstrated that it belongs to the class *Tectiliviricetes*, order *Rowavirales*, family *Tectiviridae*, genus *Alphatectivirus*. Furthermore, Akremi et al. (2020) described the first filamentous (rod-shaped) single-stranded DNA (ss DNA) phages from Tunisia specific to *E. amylovora*, currently placed in the order *Tubulavirales*, family *Inoviridae*, genus *Lineavirus*. Collectively, these findings yielded novel insights into the molecular biology, taxonomy and functional potential of *E. amylovora* phages, providing a solid foundation for their application as BCAs against FB (Gayder et al., 2023).

### 2.3 Life cycle

Most *E. amylovora* phages investigated for biocontrol potential are lytic or virulent phages, selected for their ability to reduce bacterial populations in liquid cultures and/or in plant material. During the lytic cycle a phage adsorbs to the host cell, injects and replicates its genetic material, and ultimatelly causes host cell lysis at the end of the infection cycle, resulting in the release (burst) of new infective phage progeny into the environment. The formation of clear plaques on a bacterial lawn is generally indicative of a full lytic cycle and complete host cell lysis (Clokie et al., 2011; Abedon, 2021).

In contrast, temperate phages have lysogenic life cycle, meaning that their genetic material is integrated into the host genome, as a prophage, or maintained as an episome and remains dormant until stress conditions trigger the lytic phase. A small percentage of lysogens spontaneously switch to the lytic cycle. Turbid or hazy plaque zones in the medium are commonly associated with temperate phages, because lysogenic bacteria can survive and grow within the plaque area, resulting in partial clearing rather than complete lysis (Abedon, 2021). Prophages account for genetic variability among host strains or related species in many bacteria. They can enhance the host virulence by carrying determinants for virulence, promoting the evolution and ecological fitness of bacteria during infection and protecting host bacteria from the infection of other lytic phages, which makes them of great importance (Denomy et al., 2016; Fortier and Sekulovic, 2013).

For biocontrol, strictly lytic phages are generally preferred because they rapidly replicate in and lyse target bacterial cells without integrating into the host genome. Temperate phages are unsuitable for direct application unless carefully engineered and assessed because lysogeny can alter bacterial phenotype and may contribute to horizontal gene transfer. Therefore, comprehensive genomic characterization has become an essential prerequisite for the selection of candidate phages intended for biocontrol application. This ensures the absence of integrases, lysogeny-associated regulatory genes, virulence factors, toxin genes, and antibiotic resistance determinants that could compromise safety or efficacy, because of their potential for transduction into pathogenic bacteria (Gill and Hyman, 2010; Pirnay et al., 2015; Buttimer et al., 2017).

Roach et al. (2015) investigated the prevalence of lysogeny by screening 161 *E. amylovora* isolates collected from 16 geographicaly distinct regions as well as 82 *Pantoea agglomerans* isolates from southern Ontario. Using a multiplex real-time PCR assay, the authors detected no prophages in any of the isolates, indicating the absence of lysogeny in natural populations. These findings suggest that the risk of lysogenic conversion or phage-mediated horizontal gene transfer is negligible under natural conditions. Although lysogeny has been observed under laboratory conditions, it appears uncommon in the phyllosphere and other nutrient-rich habitats such as flower washings. This evidence further supports the biosafety of employing phages and carrier bacteria in FB disease control. The same study also demonstrated that the *E. amylovora* lysogenic strain 110R, carrying prophage ΦEa35-20, was resistant to infection by closely related podovirus-like phages, but remained susceptible to a miovirus-like phage. This observation is consistent with phage-specific super infection immunity, whereby lysogens are protected against reinfection by genetically related phages, but remain susceptible to unrelated phages (Roach et al., 2015).

Akremi et al. (2020) isolated filamentous phages specific for *E. amylovora* that are neither non-lytic, nor temperate viruses. They could have advantages over lytic ones as they establish a persistent association with bacterial host cell after infection, continuously releasing progeny into the environment. During this association, the viral ssDNA is converted into a double-stranded replicative -plasmid form in the host cell. An infection by a filamentous phage can cause a loss of fitness by affecting the regulatory mechanisms of the sRNAs of *E. amylovora* and therefore attenuate the bacterial host.

### 2.4 Mechanisms of phage-host interactions

The high specificity of phage attachment is a key determinant of host range. Initial interactions between phages and their bacterial hosts are typically mediated by phage tail proteins and diverse receptors located on the cell surface. Tail structures can be highly complex, often containing additional structures like tail spikes and fibers, that mediate recognition and binding to host surface components. This adsorption machinery is the most rapidly evolving regions of tailed phage genomes (Hendrix et al., 1999) (**Table 1**).

In *E. amylovora*, host receptors include diverse surface structural components and molecules, such as outer membrane proteins, lipopolysaccharides (LPSs), capsular polysaccharides, infection-associated EPS and appendages such as pili and flagella. The extracellular matrix is an important determinant of phage-host interactions, as it can act as a physical barrier to phage adsorption on bacterial cells, while also promotes virulence, biofilm formation, host defense mechanisms and protection against desiccation (Born et al., 2014; Roach et al. 2013).

To successfully infect bacteria embedded within polysaccharide capsules or biofilms, phages have evolved mechanisms to overcome these barriers. Phages infecting *E. amylovora* often produce enzymatically active proteins involved in the crucial steps of lytic cycle enabling degradation of bacterial structures such as peptidoglycan, polysaccharides, the cell membrane, and the capsule (Gayder et al., 2023). By degrading the capsule - a key *E. amylovora*-virulence factor, these enzymes disrupt biofilm formation and expose surface receptors necessary for phage adsorption, enabling phages to reach the cell membrane, initiate infection and complete the lytic cycle (Born et al., 2011). They are commonly associated with structural components, tail spike or fiber proteins and are considered important virulence-modifying tools (Born et al., 2014; Latka et al., 2017; Abedon et al., 2017; Knecht et al., 2020).

EPS-degrading enzymes (EPSDs), including polysaccharide depolymerases, represent important phage-associated factors that can promote infection of encapsulated and biofilm-forming *E. amylovora* cells (Pires et al., 2016). By degrading extracellular polymers such as amylovoran, levan and bacterial cellulose, these enzymes weaken the protective matrix, facilitate phage access to bacterial surface receptors and contribute to biofilm disruption (Knecht et al., 2022). Because many depolymerases recognize specific surface polysaccharides, they may also shape phage host range and host specificity. In support of their functional relevance, 33 putative EPSDs have been identified in *Erwinia*- and *Pantoea*-infecting phages, most of them associated with tail structures, supporting their role in host recognition, matrix penetration and early infection events (Zlatohurska et al., 2023).

Functional studies further illustrate the relevance of EPSDs in *E. amylovora* phage biology and biocontrol. EPSD activity has been demonstrated or predicted for several phages, including phi-Ea1h, L1, Bue1, RAY, Y2 and S6 (Kim and Geider, 2000; Born et al., 2014; Knecht et al., 2018; Sharma et al., 2019). For instance, phage PhiEa1h preferentially infects encapsulated strains, suggesting the capsule-associated recognition, while expanding halos around plaques formed by PEa7 and Key indicate EPS degradation (Ritchie and Klos, 1979; Born et al., 2014; Zlatohurska et al., 2023). In phage Key, a EPSDs-encoding gene (gene 141) is located outside the structural gene cluster, indicating that EPSD functions may differ in genomic organization among related phages (Zlatohurska et al., 2023). Beyond facilitating adsorption and infection, EPSDs can also enhance phage-mediated biocontrol. The recombinant depolymerase DopL1, derived from phage Y1, enhances the infectivity and lytic activity of phage Y2 by degrading the amylovoran capsule, resulting in reduced bacterial populations on plant tissues. Similarly, cellulose has been identified as a receptor for phage S6, whose encoded cellulase facilitates host infection contributing to FB suppression (Knecht et al., 2022). In addition, phage PhiEaH2 carries *amsF* genes involved in amylovoran biosynthesis, suggesting that some phage-encoded functions may influence host physiology and pathogenicity (Kim et al., 2004). Together, these findings indicate that EPSDs are not merely accessory enzymes, but key determinants of phage infectivity, host specificity, matrix penetration, and potential efficacy in phage-based FB management.

Besides depolymerases, which primarily act outside the cell during host recognition and matrix penetration, phages also encode holins and endolysins that function mainly during the final stage of the lytic cycle. Holins permeabilize the bacterial inner membrane, enabling endolysins to access and degrade the peptidoglycan layer. This coordinated holin-endolysin system promotes efficient host-cell lysis and release of progeny phages. In *E. amylovora* phages, endolysins such as those encoded by PhiEa1h, have shown antibacterial activity in planta and may represent promising BCAs due to their rapid bacteriolytic activity and potentially low likelihood of resistance development (Ritchie and Klos, 1979). Overall, EPS depolymerases, holins and endolysins contribute to phage efficacy at distinct but complementary stages of infection: depolymerases facilitate biofilm penetration and receptor access, holins regulate access to the cell wall, and endolysins execute bacterial lysis. Their combined activities are therefore important for phage infectivity, host specificity, biofilm disruption and the development of phage- or enzyme-based strategies for FB management. Combining phages with complementary enzymatic activities and host specificities may further improve phage efficacy, highlighting the importance of host-receptor identification and functional characterization when designing effective phage cocktails (Born et al., 2011; Gayder et al., 2023).

### 2.5 Host range and diversity

Host range analysis is one of the most important criteria in the characterization and selection of phages for individual or cocktail-based applications, providing valuable insight into phage diversity, receptor specificity and the potential complementarity among phages (Kaneko et al., 2026). According to host specificity, most *E. amylovora* phages have a narrow host range, infecting only a limited subset of strains. While the high specificity enables selective targeting of *E. amylovora* with minimal disruption of the surrounding microbiota, it also represents a major limitation for field application, as individual phages often infect only a subset of pathogen populations. Consequently, comprehensive host-range analyses using genetically and geographically diverse *E. amylovora* isolates have become a prerequisite for selecting robust biocontrol candidates. A broad-host-range or polyvalent phages, which infect multiple *E. amylovora* strains and, in some cases, closely related bacteria may be advantageous for controlling genetically diverse pathogen populations. However, this also requires careful assessment of non-target effects (Svircev et al., 2006; Meczker et al., 2014; Buttimer et al., 2017). For example, some *E. amylovora* phages can also infect related *Enterobacterales*, including *Pantoea* spp., which may include beneficial epiphytes or strains used as phage carriers or BCAs (Lehman et al., 2009; Born et al., 2011; Gayder et al., 2020; Besarab et al., 2020). Therefore, candidate phages and phage cocktails should be screened against representative pathogenic strains, beneficial orchard-associated bacteria and intended carrier strains before field application.

Combining phages that recognize different bacterial surface receptors into cocktails is a widely applied approach for broadening the host range of phage preparations and improving biocontrol reliability (Abedon, 2011; Abedon et al., 2017; Born et al., 2011). This is particularly important for *E. amylovora*, whose strain-level diversity can limit the efficacy of single-phage applications. For example, some phages isolated in South Korea, such as phiEaP-8 and phiEaSP1 (Park et al., 2018; Kim et al., 2025), exhibit narrow host specificity, while others, including, pEa_SNUABM_12, pEa_SNUABM_ 47 and pEa_SNUABM_50, show broader lytic activity and the ability to cross-infect different strains within the *Erwinia* and *Serratia* genera (Kim et al., 2020). Accordingly, an improved biocontrol can be achieved by strategically selecting mixtures of *E. amylovora* phages with complementary host ranges and infection mechanisms. Recent studies support this approach. Jo et al. (2023) proposed a four-phage cocktail targeting both *E. amylovora* and *E. pyrifoliae* strains, which infected 98.91% of tested *E. amylovora* and 100% of *E. pyrifoliae* strains (Jo et al., 2023). Similarly, Born et al. (2011) developed phage cocktails composed of two virulent phages with favorable genomic profiles and complementary host ranges, resulting in significantly improved control of *E. amylovora* growth, likely due to synergistic interactions between phages. Such synergy may arise when phages target distinct surface receptors, use different infection mechanisms, or encode enzymes that modify extracellular polysaccharides and facilitate access to bacterial cells. Also combining strains with different EPS levels can broaden host range assessment. Gayder et al. (2020) showed that both geographic origin and EPS production influence phage infectivity, indicating that locally adapted phages may improve the control of regional *E. amylovora* populations.

Nevertheless, due to the dynamic nature of phage-host interactions, the sustained efficacy of phage cocktail should be monitored over time and across pathogen populations. In addition, increasing cocktail complexity also introduces challenges, including potential antagonistic interactions among phages, differential stability during storage and formulation, and difficulties in quality control and regulatory standardization. Future advances are therefore likely to rely on integrating genomic analyses, phenotypic characterization and ecological data into predictive frameworks that enable the rational design of stable and highly effective phage mixtures for field application (Kaneko et al., 2026).

Svircev et al. (2006) and Lehman (2007) studied the compatibility of *E. amylovora* phages with the saprophytic bacterium *P. agglomerans* that colonizes the same ecological niche as the pathogen, particularly the floral surfaces that serve as primary infection sites. In this approach, this compatible bacterium is used as both a carrier strain and a phage delivery system, supporting targeted delivery, improved persistence in the phyllosphere and continuous local phage replication on apple flowers (Svircev et al., 2006; Lehman, 2007; Boulè et al., 2011; Yagubi et al., 2014; Roach et al., 2015). Moreover, *P. agglomerans* can contribute directly to disease suppression through antagonistic activity, including competition for nutrients and space. The combined effects of phage-mediated bacterial lysis and bacterial antagonism create a complementary biocontrol system that can enhance disease suppression comparable to streptomycin under same experimental conditions (Svircev et al., 2006).

### 2.6 Bacterial resistance development

Because bacteria and phages are engaged in continuous co-evolution, they have developed reciprocal adaptations. Phage application imposes selective pressure that can favor the emergence of resistant bacteria, while phages may in turn adapt to maintain infectivity (Sharma et al., 2017; Hampton et al., 2020). In the process of natural selection, when a single phage is applied repeatedly, or over extended periods, bacterial populations may acquire or select for anti-phage defense strategies. These include receptor loss or modification, extracellular matrix changes, restriction-modification systems, CRISPR-Cas immunity and abortive infection mechanisms (Hyman and Abedon, 2010). Therefore, resistance should be considered as a realistic risk in phage therapy, rather than a rare, or negligible event. Under laboratory settings, resistance mutations occur more frequently than in natural environments, where microbial diversity, ecological interactions and environmental factors shape its frequency and persistence (Gomez and Buckling, 2013).

Importantly, phage resistance does not necessarily translate into increased pathogen fitness. In many systems, resistance mutations can impose trade-offs, including reduced virulence, impaired motility, altered EPS production, decreased host colonization, or increased sensitivity to antimicrobial compounds. In *E. amylovora*, phage-resistant strains exhibited increased susceptibility to kasugamycin, supporting the potential use of phage-antibiotic combinations to improve disease control, while reducing antibiotic inputs in agriculture (Kim et al., 2022). The practical impact of resistance can be further reduced through rational phage-cocktail design, rotation of phages that target different receptors, and monitoring of pathogen populations during repeated field applications (Kim et al., 2024).

## 3 Overview of major phage-based studies for FB control

Over the past two decades, numerous studies have demonstrated the potential of phages as effective BCAs against FB. Diverse *E. amylovora* phages have been isolated and characterized from different geographic regions, highlighting both their ecological diversity and potential applicability in disease management (**Table 1**). These studies have used a range of experimental systems, including *in vitro* lysis and host-range assays, immature-fruit and detached-organ models, blossom and flower assays, greenhouse or potted-plant experiments, and orchard field trials. Although these systems provide complementary evidence, they are not equivalent. Strong inhibition *in vitro* does not necessarily translate into consistent efficacy on blossoms, shoots or whole trees, where environmental factors, microbial interactions and pathogen population dynamics strongly influence phage persistence and activity (Jones et al., 2012).

### 3.1 *In vitro* lysis and host-range assays

*In vitro* assays are typically the first step in evaluating candidate phages for FB control. They provide information on lytic activity, host range, plaque morphology, bacterial reduction, phage-pathogen dynamics and preliminary safety-related traits. Early studies established the diversity of *E. amylovora* phages across different regions.

Gill et al. (2003) isolated 50 *E. amylovora* phages in Canada, while Müller et al. (2011) identified phages from North America and Germany representing different tailed-phage morphological groups. Similarly, Gašić et al. (2014) isolated seven *E. amylovora*-specific phages from irrigation water, symptomless pear leaves and apple leaves showing characteristic FB symptoms. Host-range studies against 40 Serbian *E. amylovora* strains revealed differences in lytic activity among isolates, and phage ΦEa2 was selected for further biocontrol evaluation based on its strong lytic activity.

Beyond identifying individual candidate phages, *in vitro* studies have also highlighted the importance of phage-phage and phage-bacteria interactions. Gayder et al. (2020) studied population dynamics and interactions among *E. amylovora, P. agglomerans*, and different phage combinations, including three-phage cocktails. Their results revealed complex interactions, including inter-phage competition, synergistic effects among phages, and synergy between phages and the bacterial antagonist. For example, the jumbo phage Ea35–70 was ineffective when applied alone, but improved FB control over 24 h when combined with Ea21–4 and Ea46-1-A1. However, at equal multiplicities of infection (MOIs), this phage cocktail also inhibited the growth of the bacterial carrier *P. agglomerans*. Adjusting the MOIs of Ea21-4, Ea46-1-A1, and Ea35–70 avoided this negative effect while improving pathogen control, demonstrating that cocktail composition and phage ratios are critical determinants of biocontrol performance (Gayder et al., 2020).

More recent studies have expanded the geographic scope and host-range diversity of *E. amylovora* phages. Kim et al. (2022) reported the first *E. amylovora*-specific phages from South Korea, isolated from apple and pear orchards. Based on plaque morphology, host range and genetic diversity, nine phages were assigned to two distinct clusters. All isolates were specific to *E. amylovora*, while seven also infected *E. pyrifoliae*, the causal agent of black shoot blight of pear and apple (Kim et al., 1999). This dual host range suggests their potential value for controlling both pathogens, which may coexist in the same environment (Park et al., 2022). Similarly, Jo et al. (2023) characterized four phages active against both *E. amylovora* and *E. pyrifoliae*, showing that cocktail treatments can broaden the host spectrum and induce synergistic effects. Kim et al. (2024) further demonstrated that a cocktail composed of phages Fifi044 and Fifi318 suppressed *E. amylovora* more effectively than either phage alone, under both *in vitro* and *in vivo* conditions.

Several studies have also emphasized the value of genomic and molecular characterization when selecting candidate phages. Sabri et al. (2022) reported potential of the lytic phage EP-IT22 isolated from wastewater samples in the Apulia region of Italy, as a potent antagonist of *E. amylovora*. *In vitro*, EP-IT22 lysed 94% of *E. amylovora* cells within 20h, as confirmed by viable quantitative PCR (v-qPCR) assay. Besarab et al. (2023) characterized *E. amylovora* phages isolated from soil samples in Belarus, showing variation in plaque morphology and host specificity. In addition to *E. amylovora*, some isolates also lysed *P. agglomerans* and *Pantoea ananatis*, a feature that may support phages multiplication on nonpathogenic bacterial strains for practical application and research. Seven of these phages were sequenced, and their complete genome sequences were deposited in GenBank. Xi et al. (2024) described the phage RH-42-1, isolated from FB-affected areas in Xinjiang, China, as another promising biological control candidate. The phage showed strong lytic activity and environmental resilience and genome analysis revealed genes encoding lysozyme and perforin, while no integrase, transposase, antibiotic- resistance genes, or virulence factors were detected, supporting its potential safety for biological application.

Together, these *in vitro* studies demonstrate the broad geographic and biological diversity of *E. amylovora* phages and provide an essential basis for candidate selection. However, they also show that host range, cocktail compatibility, genomic safety, and interactions with nonpathogenic bacteria must be evaluated carefully before moving toward more complex plant-based and field systems.

### 3.2 Detached-organ, immature fruit and pear-slice assays

Detached-organ and immature-fruit assays provide an intermediate experimental system between *in vitro* tests and blossoms, shoots, or whole-plant trials. By evaluating phage efficacy directly on plant tissues under controlled conditions, these assays offer a useful early indication of whether promising *in vitro* activity can translate into suppression of symptom development *in planta*.

Schwarczinger et al. (2017) isolated phages from infected fruits in Hungary and reported a 25% infection reduction in a pear slice assay. Akremi et al. (2020) demonstrated that pretreatment with a cocktail of four highly specific, bnon-lytic filamentous phages reduced symptom development in a pear-slice bioassay by 40%. Although these phages were not lytic, they impaired bhost fitness and reduced its virulence by decreasing flagellar motility, suggesting that phage-mediated attenuation of pathogenicity may also contribute to disease suppression.

Kim et al. (2022) further evaluated a combined strategy using a phage cocktail and the antibiotic kasugamycin. Five phages with complementary host ranges, active against recently isolated *E. amylovora* and *E. pyrifoliae* strains, were combined in a cocktail that was able to lyse phage-resistant strains showing decreased tolerance to kasugamycin. This phage-antibiotic synergistic effect was demonstrated both *in vitro* and on immature apple fruits, suggesting that such phage-antibiotic combinations may help reduce antibiotic inputs while limiting the emergence of resistant bacteria populations.

### 3.3 Blossom and flower assays

Blossom and flower assays are particularly relevant for evaluating FB control strategies because flowers represent a major infection site for *E. amylovora*. These assays therefore provide a biologically meaningful system for assessing whether phages can persist and function on floral tissues, where pathogen multiplication and initial infection occur.

Boule et al. (2011) showed that the use of a carrier bacterium prolonged phage persistence and enabled effective disease control at lower phage concentrations. Phages Ea1337–26 and Ea2345-6, delivered with *P. agglomerans* Eh21–5 as a carrier, reduced FB infection on pear blossoms by 84% and 96%, respectively, while the combination of Ea2345–6 and Eh21–5 reduced infection on apple blossoms by 56%. Gašić et al. (2014) further evaluated phage ΦEa2 in pear and apple blossom bioassays. Application of host-specific phage suspension either 2h before pathogen inoculation or at the time of inoculation, significantly reduced symptom development compared with the untreated control. Schwarczinger et al. (2017) also reported a 45% reduction in bacterial population in treated apple flowers, further supporting the potential of phage-based treatments on floral tissues.

### 3.4 Greenhouse and potted-plant experiments

Greenhouse and potted-plant experiments provide a higher level of biological relevance than detached-tissue assays because they evaluate disease development on intact plants under controlled environmental conditions. These systems allow assessment of phage efficacy in the context of plant architecture, tissue colonization, and more realistic pathogen spread.

*In planta* assays with the lytic phage EP-IT22 showed protection of infected pear plants, with efficacy comparable to streptomycin treatment (Sabri et al., 2022). Choe et al. (2023) evaluated pretreatment with phage FFifi106, isolated from soil in a pear orchard, for disease control in apple plants. At a MOI of 1000, the treatment reduced disease incidence to 37.0%, outperforming commercial phage-based products, such as AgriPhage-FireBlight (Kyungnong Co., Ltd., Seoul, Republic of Korea) and the antibiotic Bramycin (Farm Hannong Co., Ltd., Seoul, Republic of Korea). These findings indicate strong potential for Fifi106 as a candidate for managing *E. amylovora* infections in apple. Kim et al. (2025) evaluated biocontrol efficacy of phage phiEaSP1 against *E. amylovora* in apple seedlings. At MOI of 100, both single phage and the phage cocktail significantly reduced FB symptoms, with the cocktail providing slightly better and more sustained protection, comparable to standard antibiotic treatment.

### 3.5 Orchard field trials

Orchard field trials represent the most relevant, but also the most demanding, level of evidence for practical FB control. Under field conditions, phages must remain active despite fluctuating temperature, UV irradiation, rainfall, variable plant-surface conditions, and complex microbial communities.

Biosca et al. (2024) described the first lytic phage cocktails in Europe with effective biocontrol activity against *E. amylovora*. Such studies are particularly important because they move phage-based FB control closer to practical orchard application. However, field performance remains strongly influenced by formulation, application timing, phage persistence, compatibility with bacterial carriers or other treatments, and environmental conditions. Gdanetz et al. (2024) studied phage efficacy at in multisite field studies. Both experimental and commercial phage products, showed inconsistent efficacy (0-82.7%). Therefore, additional multi-location and multi-season orchard trials are still needed to optimize their use, confirm consistency and support registration of phage-based products.

Across studies, phage efficacy depended strongly on the experimental system, bacterial strain set, phage dose, delivery method and environmental conditions. Cocktail treatments generally improved coverage of *E. amylovora* strain diversity and reduced the likelihood of resistant populations, but consistent orchard-level control requires appropriate formulation, repeated or well-timed application and compatibility with other FB management practices.

## 4 Challenges and limitations in phage application

### 4.1 Phage stability, formulation, application and efficacy under field conditions

**Figure 2:**
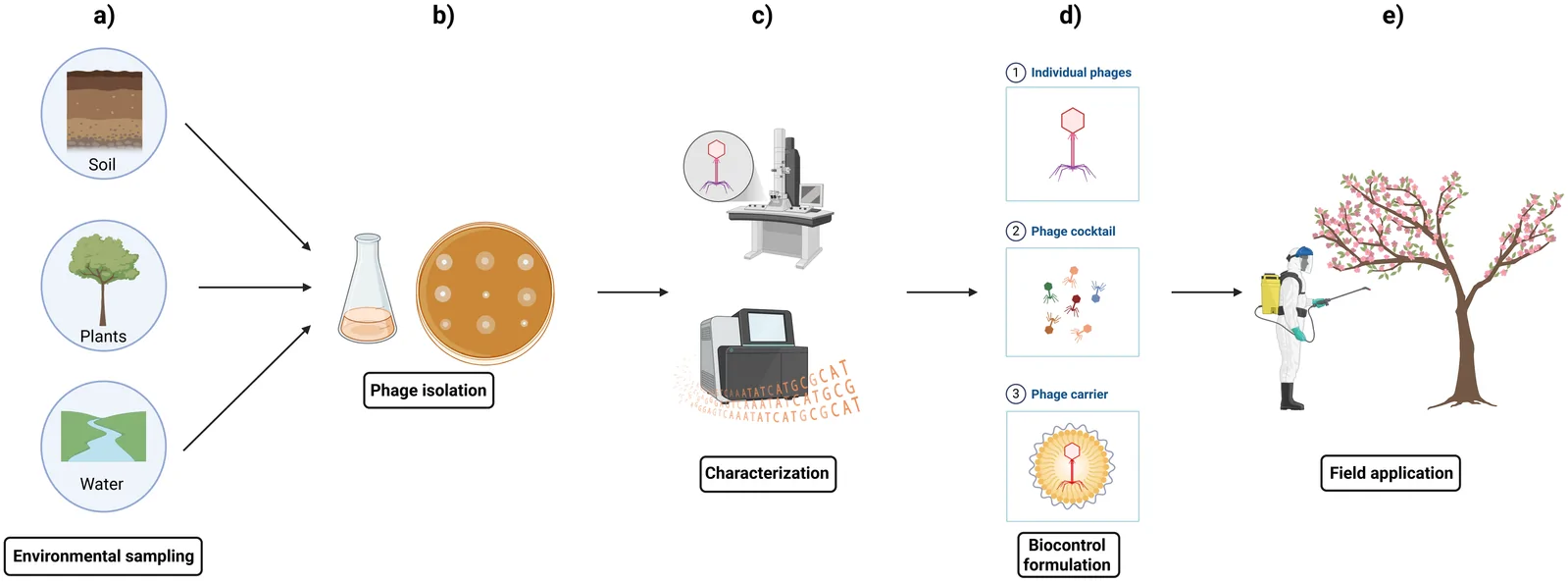
Schematic overview of the development of a phage-based biocontrol strategy for fire blight, including **(a)** isolation of **(e)** amylovora-specific phages, **(b)** their characterization, **(c)** development of formulation strategies and **(d)** field application. Created in BioRender.

The development of effective phage therapy for FB requires the isolation of highly lytic phages, their detailed characterization, formulation and delivery optimization, and validation under conditions that reflect commercial orchard environments (**Figure 2**).

Phage stability and survival after field application are one of the major challenges (Balogh et al., 2018). As protein-based biological agents, phages are highly sensitive to a range of abiotic factors, resulting in limited persistence and short residual activity on plant foliage, which can significantly reduce their efficacy under field conditions (Iriarte et al., 2007). In the phyllosphere, they are exposed to ultraviolet (UV) radiation, high temperature, desiccation, pH and relative humidity variation, all of which can reduce short-term persistence on the plant surfaces (Ehrlich et al., 1964; Balogh et al., 2003; Iriarte et al., 2007; Holtappels et al., 2021; Gdanetz et al., 2024). UV radiation is particularly limiting because it can directly damage phage nucleic acids and rapidly reduce phage viability. Therefore, UV-protective formulations are essential for improving phage persistence and efficacy under orchard conditions (Balogh et al., 2003; Iriarte et al., 2007).

Successful orchard application also requires spatial and temporal overlap between active phage particles and susceptible *E. amylovora* populations. Formulations must protect virions from UV light, desiccation and temperature extremes, while spray programs must provide adequate canopy and blossom coverage. Application timing is equally important: evening or early morning treatments can reduce UV-induced inactivation, and integration with disease-forecasting systems may help target applications to high-risk bloom periods and avoid unnecessary treatments (Balogh et al., 2003; Iriarte et al., 2007). In addition, monitoring pathogen populations could support adjustment of cocktail composition and phage rotation during repeated use.

Several formulation strategies have been shown to improve phage survival in the phyllosphere. Additives such as skim milk, sucrose, and UV-absorbing phenolic compounds, can reduce phage degradation and prolong persistence on plant surfaces (Balogh et al., 2003; Iriarte et al., 2007; Born et al., 2015; Gašić et al., 2018). For *E. amylovora* phages specifically, Born et al. (2015) showed that peptone (50 mg/ml) and dark-colored plant extracts such as beetroot, carrot and red pepper juices, increased the survival of phage Y2 by nearly two log units after UV exposure. Jo et al. (2023) further demonstrated the protective effect of polysorbate 80 and kaolin under UV irradiation during 6 h with 4.5% kaolin providing optimal UV protection for the tested *E. amylovora* phages. Most phages formulated with 4.5% polysorbate 80 and kaolin persisted on leaf surfaces for more than two weeks after application, highlighting the potential of formulation to enhance field performance.

However, Gdanetz et al. (2024) showed that the addition of UV protectants like kaolin clay, peptone and carrot juice to spray formulations does not consistently translate into improved field efficacy, indicating that environmental conditions remain a key determinant of treatment performance.

Recent preliminary work by Prokić et al. (2025) and Ivanović et al. (2025) also evaluated the protective effect of natural compounds such as kaolin, riboflavin and skimmed milk, on vitality of selected *E. amylovora* phages after UV exposure *in vitro*. These results suggested improved UV-persistence of formulated phages compared with non-formulated controls. However, these findings should be confirmed in further peer-reviewed studies and under greenhouse or field conditions before firm conclusions can be drawn.

Overall, phage formulation should be viewed as a relevant component of phage-based FB management, not as a secondary technical step. Protective additives, appropriate timing, disease-risk forecasting, spray coverage, and monitoring of pathogen populations all need to be integrated to improve the consistency of phage performance under variable orchard conditions.

### 4.2 Commercially registered phage-based biopesticides for FB control

Regulatory approval for phage-based treatments in agriculture is still evolving, and further research is required to demonstrate their efficacy, environmental safety and manufacturing consistency. Several phage-based products are now commercially available and have shown successful control of *E. amylovora* under field conditions (**Table 3**). However, their registration status and permitted applications vary among countries and may change over time. Therefore, product names, registration numbers, approved target crops, active phage composition and application should be verified against current authority labels or manufacturer documentation before being described as commercially registered plant protection products. In the US, registration information is available through the Environmental Protection Agency (EPA, 2026), In Europe, product authorization is generally assessed through national registration databases and manufacturer documentation. For example, the Hungarian company Enviroinvest developed Erwiphage formulation for FB management, including Erwiphage Plus and Erwiphage Forte. Published reports indicate that these products contain *E. amylovora*-specific phages as active BCAs for use on apple (Schwarczinger et al., 2017).

**Table 3:** Phage-based products and initiatives developed for FB biocontrol.

Product/initiative	Product registration status	Active phage composition	Target crop	Application site
AgriPhage – Fire Blight (Omnilytics, Inc.)	Active U.S. EPA registration as a microbial biopesticide/bactericide (EPA Reg. No. 67986-8.	Lytic bacteriophage cocktail; minimum 5.0 × 10¹² PFU/L	Apple and pear	Foliar treatment, irrigation supply systems (water) treatment, areal treatment
Erwiphage PLUS (Enviroinvest Corporation Kft.)	National/emergency authorization in Hungary	Two-phage cocktail (PhiEaH1 and PhiEaH)	Pome-fruits	Foliar treatment
DCM PEA-02^®^	Pending full EU approval; emergency authorizations granted in multiple EU countries (Belgium, Portugal, Spain, Germany, France, and Greece)	Lytic bacteriophage cocktail	Apple and pear	
PhageFire	Development-stage/pre-commercial EU biopesticide	Lytic bacteriophage cocktail	Apple, pear, and quince	Foliar treatment

## 5 Conclusions and future perspectives

The available literature shows that phages with high stability, strong lytic activity and broad efficacy against diverse *E. amylovora* strains could represent a relevant component of integrated FB management strategy (Kim et al., 2022; Ibrahim et al., 2024). Their main advantages include high host specificity, the capacity to reduce bacterial populations through lysis, and compatibility with other disease-control strategies. However, these advantages should be evaluated for each phage or cocktail, because host range, persistence, off-target effects and ecological behavior can differ substantially among phages, phage cocktails and orchard environments.

As biopesticide, phages may be compatible with organic or low-residue production systems provided that their use is permitted by national or regional registration frameworks. Nevertheless, the development and registration of phage-based products are not necessarily simpler or less costly than those of other biocontrol agents. Each product must demonstrate efficacy, environmental safety, manufacturing consistency, formulation stability and quality control for the intended crop, pathogen, and region. In addition, large-scale phage production remains a technical challenge, requiring reliable host-production systems, contamination control, genomic safety assessment, and standardized formulations capable of maintaining phage viability during storage, transport, and field application. Despite many successful experimental studies, the practical application of lytic phages for FB control remains limited. Achieving consistent field performance is still a major challenge, because phage activity is strongly influenced by environmental factors, host susceptibility, pathogen strain diversity, phage persistence on plant surfaces and off-target effects (Gdanetz et al., 2024). Potential safety issues, including particularly the emergence of phage resistance, or unintended effects on non-target microbiota, further emphasize the need for a deeper understanding of the molecular and ecological interactions among phages, *E. amylovora*, and the plant-associated microbiome.

Key knowledge gaps remain before phage-based FB control can be used reliably at commercial scale. Several priorities should guide future research. These include multi-location and multi-season validation under diverse orchard conditions; improved understanding of phage persistence on blossoms, shoots and fruit surfaces; optimized formulations, delivery systems and application timing; phage resistance monitoring and associated fitness costs; assessment of non-target effects on beneficial orchard-associated epiphytes and carrier strains; harmonization of regulatory pathways; and integration of phage treatments with disease-forecasting systems and existing biological or chemical control programs. Addressing these challenges will be essential for commercially applicable phage-based strategies for sustainable FB management.
